# Accurate Preoperative and Intraoperative Evaluation Reduces Surgical Costs and Patient Invasiveness in Ventriculoperitoneal Shunt Revision

**DOI:** 10.7759/cureus.62334

**Published:** 2024-06-13

**Authors:** Marina Takahashi, Taijun Hana, Shota Tanaka, Nobuhito Saito

**Affiliations:** 1 Department of Neurosurgery, The University of Tokyo Hospital, Tokyo, JPN; 2 Department of Neurosurgery, Saitama Medical Center, Saitama Medical University, Saitama, JPN; 3 Department of Neurosurgery, Okayama University Graduate School of Medicine, Dentistry, and Pharmaceutical Sciences, Okayama, JPN

**Keywords:** medical economy, surgical cost, hydrocephalus, shunt revision, ventriculoperitoneal shunt

## Abstract

The ventriculoperitoneal (VP) shunt is one of the most common surgical procedures in neurosurgery, frequently resulting in malfunctions. Shunt malfunctions, which can include mechanical failure, obstruction, infection, or disconnection, occur in a significant percentage of patients, often necessitating multiple revisions. These revisions can lead to increased healthcare costs due to additional surgeries or treatments. Therefore, addressing the economic impacts of these revisions is crucial.

Our report presents a cost-effective approach to shunt revisions, demonstrated through a case study of an 82-year-old woman with hydrocephalus. Although initially treated with a VP shunt, she required a revision after six years due to shunt malfunction. Through comprehensive preoperative and intraoperative evaluations, including a shuntogram with iodine contrast and meticulous examination, we identified the cause of malfunction as a connective tissue sac blocking the peritoneal catheter. The surgery involved flushing the catheter lumen with saline to confirm the obstruction and careful removal of the obstructive tissue. This accurate diagnosis facilitated a minimally invasive revision, enabling the reuse of existing shunt components and avoiding the need for new devices, thus reducing costs and surgical invasiveness.

Our study serves as a call to action for healthcare providers and surgeons to consider more cost-effective and patient-friendly approaches in managing VP shunt malfunctions, ultimately benefiting both the healthcare system and the patients it serves.

## Introduction

In the United States, approximately 30,000 ventriculoperitoneal (VP) shunt surgeries are performed annually, with 41% being revisions due to shunt malfunctions [1-3]. Shunt malfunction poses a significant concern in VP shunting, particularly evident in the failure rate of 15-23% in adults and a higher frequency in pediatric cases, where 27% undergo revisions [4,5]. This issue extends beyond clinical implications, presenting a substantial economic challenge in health care. On average, each VP shunt revision incurs a cost of $37,543, contributing to the $100 million spent annually on shunt placements in the United States, almost half of which is dedicated to revisions [6,7]. Addressing the economic impact of shunt revisions is thus critical. Our report highlights a case where thorough preoperative and intraoperative evaluations notably decreased the costs and invasiveness associated with shunt revision surgery, offering a strategy that could significantly reduce the global economic burden of shunt-related procedures.

## Case presentation

Patient

**Figure 1:**
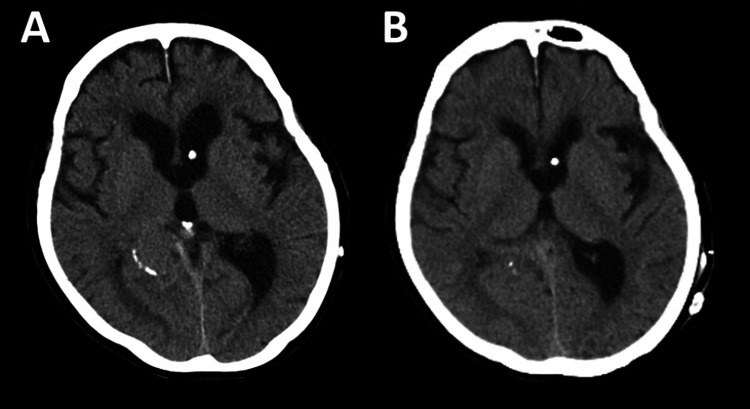
CT images (A) Preoperative CT. (B) Postoperative CT.

Our case is an 82-year-old woman who presented with incoherent speech, forgetfulness, and walking difficulties. An MRI scan indicated ventricular enlargement, with an Evans index of 0.35. The Evans index is defined as the ratio of the maximal width of the frontal horns to the maximal internal diameter of the skull, with values >0.30 representing ventricular enlargement [8]. She was diagnosed with normal pressure hydrocephalus and underwent VP shunt placement with the Sophysa Polaris system (Sophysa, Crown Point, IN), set at a pressure of 150 mm H2O. This procedure initially improved her gait disturbances. However, six years post operation, she began experiencing a gradual recurrence of her walking difficulties. A CT scan (Figure 1) revealed an enlargement of the ventricles (Figure 1A). Despite multiple adjustments to the shunt valve pressure, her symptoms persisted, accompanied by continued ventricular enlargement. Shunt malfunction was suspected, and a shunt revision was deemed necessary. In preparation for and during the surgery, detailed evaluations were conducted to ensure minimal invasiveness for the patient.

Preoperative evaluation

**Figure 2:**
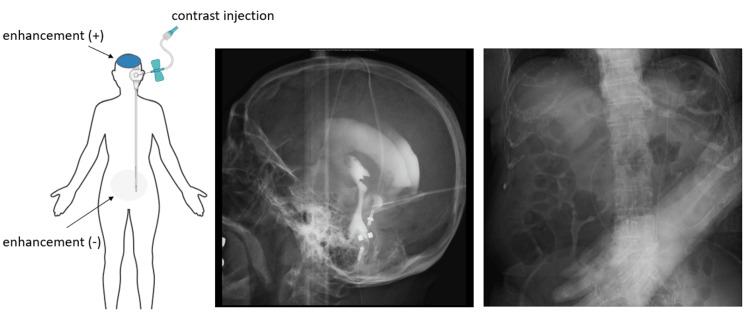
Preoperative shuntogram (Left) Schematic diagram showing the smooth flow of the contrast agent into the ventricles, but no progression toward the abdominal cavity. (Middle) Fluoroscopic image of the ventricles, highlighting their structure and the presence of the contrast agent. (Right) Fluoroscopic image of the abdominal side, illustrating the absence of contrast agent in this area.

To accurately determine the necessary reconstruction part of the VP shunt during surgery, we utilized a shuntogram with iodine contrast (Isovist injection 240, Bayer Japan, Osaka, Japan). Successful, smooth cerebrospinal fluid (CSF) collection was achieved through reservoir puncture, suggesting the patency of the ventricular catheter. Once the contrast agent was injected into the reservoir, we obtained clear imaging of the bilateral ventricles and the third ventricle, but neither the peritoneal catheter nor the abdominal cavity was enhanced even after vigorous pumping of the reservoir (Figure 2). Using the affiliated magnetic device, the valve's function was thoroughly checked, confirming that there were no abnormalities with the valve itself. A subsequent CT scan also confirmed the absence of the contrast agent in the abdominal cavity. These findings led us to conclude that the malfunction was not due to valve failure, but rather to an obstruction on the abdominal side of the shunt. The CSF collected during the shuntogram showed no signs of infection, and the culture was also negative.

Intraoperative evaluation and procedure

**Figure 3:**
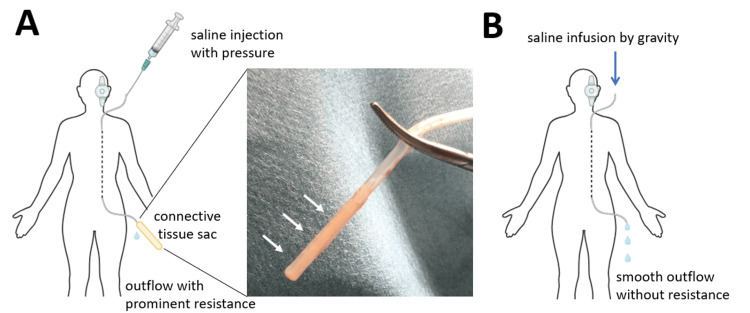
Schematic diagram of cerebrospinal fluid flow changes during surgery (A) The peritoneal catheter's tip was obstructed by a connective tissue sac (arrows), blocking saline outflow. (B) Following removal of the connective tissue sac, saline flowed smoothly from the peritoneal catheter.

We initially exposed only the valve portion and confirmed that there were no foreign objects or structural defects within the valve. Then we severed the peritoneal catheter near the valve's distal side and flushed it with saline only to find impeded flow, indicating a possible obstruction within the peritoneal catheter. Consequently, we reopened the abdominal incision to carefully extract the peritoneal catheter that was positioned within the abdominal cavity. We discovered a connective tissue sac completely enveloping the side holes at the peritoneal catheter's tip, which was impeding the outflow of CSF (Figure 3A and Video 1). The tip of the peritoneal catheter was trimmed and no further blockage was ensured through flushing and infusion of saline by gravity (Figure 3B and Video 2). Then, we reconnected the peritoneal catheter to the valve and repositioned it in the abdominal cavity. This procedure was completed with a minimal number of skin incisions and without the need for any new devices, preventing additional brain procedures and the re-tunneling of the peritoneal catheter, thus minimizing surgical invasiveness.

**Video 1:** Saline outflow before connective tissue removal During the saline injection, strong resistance was encountered, resulting in only a small amount of fluid being expelled from the distal end of the catheter.

**Video 2:** Saline outflow after connective tissue removal After the removal of the connective tissue sac, the saline flowed effortlessly, driven solely by gravity, with a steady drip observed from the distal end of the peritoneal catheter.

Postoperative course

We closely monitored the size of the ventricles with serial CT scans and appropriately adjusted the shunt pressure (Figure 1B). Subsequently, the patient showed a significant improvement in her performance status, and her neurological condition returned to its state prior to the shunt malfunction.

## Discussion

We experienced a case of VP shunt malfunction requiring shunt revision due to occlusion at the peritoneal catheter's tip. Clearly identifying the shunt occlusion site both preoperatively and intraoperatively allows us to minimize the use of new devices, leading to less invasiveness and cost reduction. A shuntogram can be performed safely using a non-ionic, low-osmolarity iodine contrast agent. Meticulous imaging evaluation generally allows for accurate identification of the cause of shunt malfunction. During surgery, injecting saline into the catheter lumen and checking flow resistance can more precisely locate where the shunt needs to be revised. Such detailed evaluation often prevents the need for opening new devices, resulting in a significant reduction in surgical costs. It also helps to minimize the number of skin incisions needed and prevent additional brain procedures, leading to less invasiveness for the patient.

**Figure 4:**
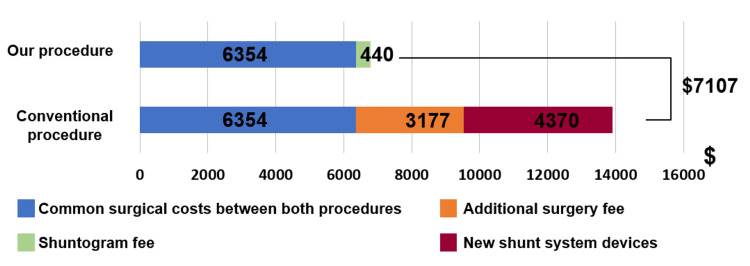
The cost differences between the conventional procedure and the procedure used in this case Conventional shunt revision takes about 135 minutes, whereas our method shortens the process by an average of 45 minutes, anticipating a cost reduction of $3177. Additionally, our procedure does not use new shunt devices, thus saving another $4370 on device costs. As illustrated in this figure, even if the cost of the shuntogram is added, our procedure is expected to reduce overall costs.

We here compare the costs of a conventional shunt revision and our procedure. In our method, the only additional cost compared to the conventional method is the shuntogram. In this case, the shuntogram was performed promptly along with other tests during the short waiting period from admission to the day of surgery and therefore did not extend the hospital stay. Figure 4 illustrates the cost differences between a conventional shunt revision and the costs incurred in our procedure. According to a previous report, the estimated cost of one shuntogram at a university hospital in the United States is a total of $440 [9]. According to the price information of medical vendors (CODMAN Ventricular Catheter: $175.00, CODMAN Peritoneal Catheter: $200.97, GeoSurgical, Clearwater, FL; CODMAN CERTAS Plus Programmable In-line Valve: $3,995.00, SUTURES, Mokena, IL), the total price of the shunt system in the United States was estimated to be $4,370. Considering the average surgery time for a typical shunt revision at our facility, the surgical and anesthesia time was estimated to be 135 minutes. Since our procedure was 45 minutes shorter on average, the surgical and anesthesia time using our method was calculated to be 90 minutes. This difference is reasonable, considering that procedures like inserting a ventricular catheter or tunneling a new peritoneal catheter are not required. A study of 100 facilities in the United States reports that the average operating room charge is $62 per minute [10]. Additionally, a report indicates that the total cost of anesthesia care in the United States is $8.62 per minute [11]. Based on these two reports, we calculated the common surgical costs between both procedures and the additional surgery fee for the conventional procedure. The common surgical cost is calculated to be $6,354 ($70.6 × 90 minutes). The additional surgery fee for the conventional procedure is calculated to be $3,177 ($70.6 × 45 minutes). As illustrated in this figure, even when accounting for the cost of the shuntogram, our procedure is expected to result in substantial cost savings of over $7,000.

A potential topic for discussion is the feasibility of reusing the pre-existing shunt system. Previous reports indicate that the "durability" of a shunt system does not significantly change, whether it is completely or partially replaced [12]. Shunt device manufacturers have not specified a particular lifespan for either shunt device: peritoneal catheter, ventricular catheter, or valve. These components can generally remain in place as long as they function properly, similar to non-electrical implantable devices such as artificial joints. Regarding the valves specifically, it is reported that 60-80% of them remain functional even 10 years after implantation [13]. It is considered acceptable to reuse a part of the shunt system for a certain period unless the surgeon discovers an obvious sign of deterioration in the shunt system during surgery.

As a limitation, it should be noted that this study is a case report introducing our procedure, and it does not include detailed comparative examinations such as statistical analyses of multiple cases.

## Conclusions

Appropriate, preoperative examination with a shuntogram and systematic, intraoperative evaluation of the cause of malfunction not only contribute to minimizing surgical invasiveness for patients but also to reducing surgical costs in the treatment of shunt failure, the common complication associated with VP shunts.
